# Heterologous Amyloid Seeding: Revisiting the Role of Acetylcholinesterase in Alzheimer's Disease

**DOI:** 10.1371/journal.pone.0000652

**Published:** 2007-07-25

**Authors:** Létitia Jean, Benjamin Thomas, Abdessamad Tahiri-Alaoui, Michael Shaw, David J. Vaux

**Affiliations:** 1 Sir William Dunn School of Pathology, University of Oxford, Oxford, United Kingdom; 2 Central Proteomics Facility, Sir William Dunn School of Pathology, University of Oxford, Oxford, United Kingdom; Massachusetts General Hospital & Harvard Medical School, United States of America

## Abstract

Neurodegenerative diseases associated with abnormal protein folding and ordered aggregation require an initial trigger which may be infectious, inherited, post-inflammatory or idiopathic. Proteolytic cleavage to generate vulnerable precursors, such as amyloid-β peptide (Aβ) production via β and γ secretases in Alzheimer's Disease (AD), is one such trigger, but the proteolytic removal of these fragments is also aetiologically important. The levels of Aβ in the central nervous system are regulated by several catabolic proteases, including insulysin (IDE) and neprilysin (NEP). The known association of human acetylcholinesterase (hAChE) with pathological aggregates in AD together with its ability to increase Aβ fibrilization prompted us to search for proteolytic triggers that could enhance this process. The hAChE C-terminal domain (T40, AChE_575-614_) is an exposed amphiphilic α-helix involved in enzyme oligomerisation, but it also contains a conformational switch region (CSR) with high propensity for conversion to non-native (hidden) β-strand, a property associated with amyloidogenicity. A synthetic peptide (AChE_586-599_) encompassing the CSR region shares homology with Aβ and forms β-sheet amyloid fibrils. We investigated the influence of IDE and NEP proteolysis on the formation and degradation of relevant hAChE β-sheet species. By combining reverse-phase HPLC and mass spectrometry, we established that the enzyme digestion profiles on T40 versus AChE_586-599_, or versus Aβ, differed. Moreover, IDE digestion of T40 triggered the conformational switch from α- to β-structures, resulting in surfactant CSR species that self-assembled into amyloid fibril precursors (oligomers). Crucially, these CSR species significantly increased Aβ fibril formation both by seeding the energetically unfavorable formation of amyloid nuclei and by enhancing the rate of amyloid elongation. Hence, these results may offer an explanation for observations that implicate hAChE in the extent of Aβ deposition in the brain. Furthermore, this process of heterologous amyloid seeding by a proteolytic fragment from another protein may represent a previously underestimated pathological trigger, implying that the abundance of the major amyloidogenic species (Aβ in AD, for example) may not be the only important factor in neurodegeneration.

## Introduction

Several human neurodegenerative syndromes, such as Alzheimer's, Parkinson's, Huntington's and Prion diseases, are thought to possess an underlying common pathological mechanism in which protein misfolding leads to protein aggregation and polymerization. The polymerization process results in the formation of amyloid fibrils with a cross-β sheet fold that deposits and accumulates in the brain. Amyloid fibrilization is a multistep process characterized by an energetically unfavorable formation of nuclei (lag phase) followed by cooperative amyloid elongation. In the case of Alzheimer's disease (AD), the extracellular deposition of amyloid-β-peptide (Aβ) in senile plaques and intracellular deposition of hyperphosphorylated Tau in neurofibrillary tangles are characteristic of the pathology [1]. Aβ is a 40 to 43 amino acid peptide resulting from paired endoproteolysis of the β−amyloid precursor protein (APP)[2], [1]. Recent attention has focussed on Aβ catabolism to understand the mechanisms leading to its excessive accumulation during AD. A number of unrelated proteases were identified, among which insulysin (IDE) and neprilysin (NEP) are undoubtedly involved in Aβ clearance [3], [4]. IDE is a thiol zinc metalloprotease found in the brain and located mainly in cytosol [5], [6], [7]. IDE cleaves a broad range of peptides and has been proposed to be an amyloid scavenger recognizing structural β-rich folds found in amyloid forming peptides (e.g. insulin and Aβ)[4]. A genetic linkage was found between the chromosome 10q locus encoding IDE and onset of AD [8]. In IDE deficient mice, cerebral levels of Aβ are increased [5], [7], conversely mice over-expressing IDE and APP exhibited decreased Aβ levels, reduced plaque burden and protection from premature death [6]. NEP is also a zinc metalloprotease found in the brain; it cleaves on the amino side of hydrophobic residues in a variety of peptides (e.g. substance P and enkephalin)[9]. NEP localization at the plasma membrane makes it a candidate for degradation of extracellular Aβ [9]. NEP deficiency and over-expression studies in mice gave comparable results to those for IDE [3], [6]. Whereas IDE was found to degrade only soluble monomeric Aβ, NEP can hydrolyze both monomeric and oligomeric Aβ [10], [11].

Although Aβ is a key player in the pathology associated with senile plaques, other proteins such as cholinesterases have been implicated [12], [13], [14], [15]. The evidence is as follows; both human acetylcholinesterase (hAChE) and butyrylcholinesterase (hBuChE) are associated with senile plaques and both the pattern of hAChE oligomerisation and its enzymatic activity are altered in brain areas affected by AD [12], [13], [16], [14], [17]. BuChE inhibitors were shown to reduce level of APP and also to improve cognitive function in patients with moderate AD [18], [19]. Whereas hAChE activity diminishes in the cortex of AD patients, hBuChE activity remains unchanged or increases [20], [13], [14] and it has been proposed that hBuChE acts as a substitute for hAChE when hAChE is impaired [21]. However, the role of hBuChE in normal or AD brains remains unclear. Various studies suggest that hAChE promotes Aβ fibrilization and deposition in pathological aggregates [22], [23] but the mechanism remains unknown. Moreover, double transgenic mice expressing human APP (hAPP) and hAChE developed earlier disease than single transgenic hAPP mice, accompanied by increased plaque deposition and pathology [23]. Neurodegenerative diseases associated with abnormal protein folding and aggregation are nucleation-dependent, which involves a slow and unfavorable nucleation phase (known as the lag phase) during which monomers associate to form ordered oligomeric nuclei, an elongation phase during which the nuclei exceed a threshold size, become stable and monomers can be added favorably to them, and finally a plateau phase in which the monomer concentration falls below the threshold aborting further fibril extension [24]. Because of this nucleation-dependency, neurodegenerative diseases may require an initial trigger. This trigger may involve different pathological pathways including the effects of molecules acting as pathological chaperones, as well as seeding events (defined as involvement of exogenous nuclei to bypass the slow nucleation event), both homologous and heterologous. In the case of heterologous seeding, the seed originates independently of the molecular species that will make up the bulk of the accumulating misfolded material. Along with hAChE, other molecules have been shown to enhance the nucleation phase of Aβ fibrilization. For example, glycosaminoglycans and membrane glycolipids can mediate Aβ aggregation [25], [26]. Thus, one may postulate that the subtle effect of these ‘secondary’ molecules might be heterologous seeding of Aβ, which could represent one of the trigger for more severe Aβ pathology during AD.

**Figure 1:**
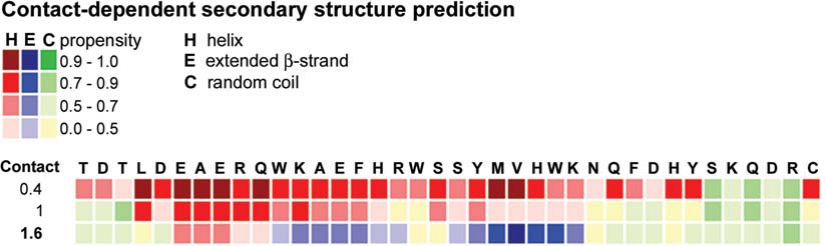
Secondary structure propensity of T40 as predicted by hidden β-propensity method (available at http://opal.umdnj.edu). Propensities for helices (red squares), β-strands (blue squares) and random coil (green squares) are presented numerically using a 0-1 scale, with low values indicating zero to low propensity and high values indicating high propensity to near certainty.

The non-amyloidogenic and α-helical C-terminal oligomerisation domain of hAChE (T40, AChE_575-614_) [27], [28], [29] contains a region that shares homology with Aβ. Computational identification of non-native (hidden) β-strand propensity in protein sequences had predicted the minimal amyloidogenic fragments for Aβ and α-synuclein [30]. When applied to T40, a short and unique predicted conformational switch region (CSR, from W_585_ to K_599_) with high propensity for conversion to non-native (hidden) β-strand was identified (Figure 1)[31], [30], with a strong propensity for conversion to β-strand for the sequence Y_594_MVHWK_599_ and A_586_EFHR_590_ more weakly. A peptide synthesized to include this CSR region (AChE_586-599_) adopts a β-sheet conformation and self-assembles into amyloid fibrils, a structure associated with AD plaques [31], [30]. AChE_586-599 _was previously identified as a region of high hidden β-propensity by the independent computational study that predicted the Aβ and α-synuclein amyloidogenic fragments. The mechanisms that could trigger such a conformational switch in this region are unknown but are worth seeking since they could represent the connection between AChE and increased Aβ fibril formation during AD pathogenesis. Proteolytic processes could liberate fibrilogenic peptides (CSR-like) from the non-amyloidogenic and α-helical C-terminus of AChE that is exposed in the monomer and implicated in tetramer formation [32]. The plausibility of such a mechanism is reinforced by the observation that hydrophilic monomers of bovine brain AChE are not reactive with an antibody raised against the extreme C-terminus of the T40 domain, consistent with C-terminal truncation events [33]. Moreover, the normal reactivity of the AChE tetrameric form with this antibody is lost after limited proteolysis of the tetrameric species, suggesting that the T40 domain remains vulnerable to proteolysis even in assembled tetramers [33]. Candidate proteases include IDE and NEP, which are known to be present and active in the extracellular space of the brain and are already clearly implicated in processing of amyloidogenic peptides in the central nervous system.

In this study, we examined the activity of IDE and NEP on formation of relevant β-sheet molecular species from the non-amyloidogenic and α-helical T40 fragment of AChE and degradation of pre-assembled β-sheet oligomers. IDE cleaved both non-amyloidogenic T40 and amyloid forming AChE_586-599_, whereas NEP only cleaved the AChE_586-599_ substrate. Digestion of the non-amyloidogenic and α-helical T40 by IDE triggered the formation of β-structures that formed amyloid precursors (oligomers) and generated surface-active CSR species (detergent-like), which seeded Aβ fibrilization by reducing the lag phase and enhancing the rate of amyloid elongation. The heterologous seeding of Aβ by IDE-mediated amyloidogenic hAChE fragments may offer an explanation for the implication of hAChE in the extent of Aβ deposition in the brain [34]. Aβ heterologous seeding by proteolytic fragments from another abundant CNS protein may also represent a previously undescribed pathological trigger, in which the abundance of Aβ may not be the only important factor in AD.

## Results

### T40-degrading activity of IDE

**Figure 2:**
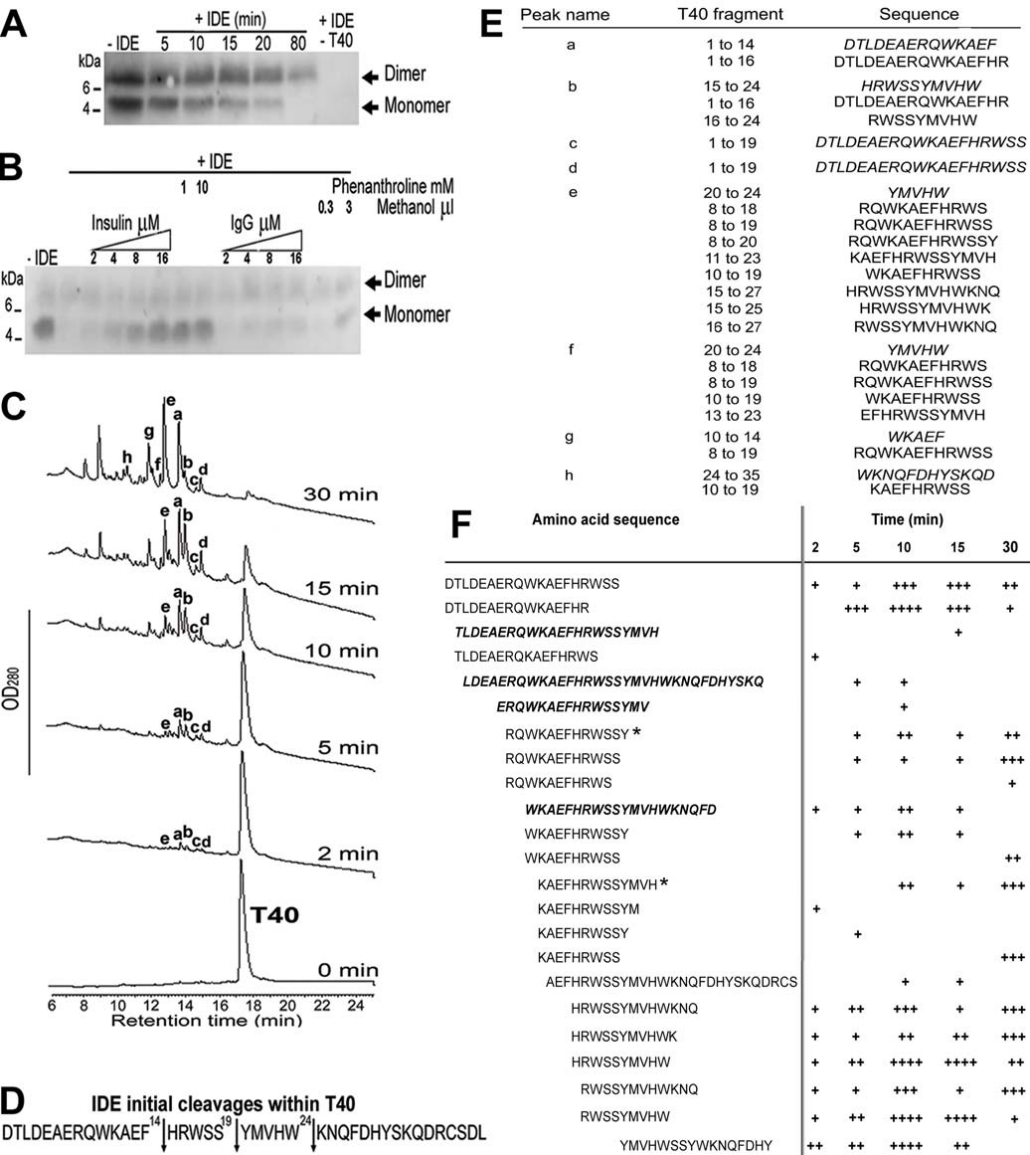
T40-degrading activity of IDE. (A) IDE degrades non-amyloidogenic and monomeric T40. 16 µM T40 was incubated with or without 22 nM IDE (37°C) and the reaction stopped as indicated. (B) Specificity of IDE activity. 16 µM T40 was incubated (37°C, 90 min) with no IDE, with 22 nM IDE or with 2–16 µM insulin, 2–16 µM IgG, 1 and 10 mM 1,10-phenanthroline, or 0.3 and 3 µL methanol. For (A) and (B), digestion products were resolved (10% Tris-Tricine SDS-PAGE), electro-blotted onto nitrocellulose and probed with KD69 (specific for the T40). Marker proteins are indicated. *Arrows* indicate the positions of T40 monomers and dimers. (C) 60 µM T40 was incubated with or without 50 nM IDE (37°C) and products separated by RP-HPLC (peaks annotated a–h). (D) Positions of IDE initial cleavages (*arrows*) within T40 (2 min digestion). (E) Identity of the major peptides (italics) and of CSR species in peaks a–h, analyzed by MS. (F) The relative abundance of CSR species was determined with reference to an internal standard on the MS spectra and is displayed as arbitrary units. ‘+’, 1 to 25 arbitrary units; ‘++’, 26 to 100; ‘+++’, 101 to 250; and ‘++++’, >250. Potential precursors of CSR species are italicized and shown in bold. ‘*’ indicates CSR species that were not present after a 2 hour digestion of T40 by IDE.

We examined the effect of proteolytic processes on the non-amyloidogenic and α-helical C-terminus of AChE (T40) that is exposed in the monomer and implicated in tetramer formation [32]. The ability of IDE to digest T40 is depicted in the western blot probed with a rabbit anti-T40 antiserum (KD69 antiserum)(Figure 2A). In the absence of IDE, the 5 kDa T40 migrates as monomers and dimers. In the presence of IDE, monomeric T40 was progressively digested with complete disappearance by 80 min incubation, whereas a proportion of the dimeric form remained undigested. The specific activity of IDE on T40 was demonstrated by a dose dependent inhibition with insulin (a natural IDE substrate) or 1,10-phenanthroline (a zinc metalloprotease inhibitor)(Figure 2B). Complete inhibition was achieved at equimolar levels of insulin (16 µM), which did not occur with an irrelevant protein (IgG). The 1,10-phenanthroline was prepared in methanol, which addition to the IDE reaction did not affect degradation of T40.

To determine a full map for IDE cleavage of T40, a temporal series of products were analyzed by mass spectrometry (MS) and reported based upon T40 numbering (Asp^1^ to Leu^40^). After 2 min incubation, four major products (a–d) were visible and corresponded to initial cleavages between Phe^14^-His^15^, Ser^19^-Tyr^20^, and Trp^24^-Lys^25^ (Figures 2C and 2D). As digest time increased, more products appeared with peaks a and b dominating up to 15 min before peak e became the prominent product at 30 min, coinciding with near complete digestion of T40. The progression and regression of peaks a–d suggests that after digestion at the primary positions, T40 fragments are serially digested to generate other peptides. A complete digestion map of T40 by IDE after 30 min incubation is available as supporting information (Figure S1). Peaks b to h contained significant amounts of CSR species (Figure 2E) along with other identified peptides. Peaks b, e and f contained species encompassing the sequence YMVHW with high propensity for conversion to β-sheet as major IDE cleavage products. Peak e, the major peak at 30 min, also contained a peptide almost identical to the previously studied AChE_586-599_ peptide (KAEFHRWSSYMVH versus AEFHRWSSYMVHWK). Analysis of peaks a–h in all the incubation times revealed that CSR species appeared very early during digestion (from 2 min) with the number and abundance increasing with time (Figure 2F). Species containing the high-β propensity sequence YMVHW appeared to be the most abundant CSR species identified during T40/IDE digest (Figure 2F, bottom of the table). Potential precursors to CSR species were detected, which increased and disappeared, coincident with the appearance of smaller peptides (e.g. WKAEFHRWSSYMVHWKNQFD versus HRWSSYMVHWK). Almost all CSR species, except RQWKAEFHRWSSY and KAEFHRWSSYMVH, were still present after a longer exposure to IDE (2 hours), indicating that the CSR species are resistant to and can survive further digestion by IDE (Figure 2F).

### IDE digests monomeric and oligomeric species of AChE_586-599_

**Figure 3:**
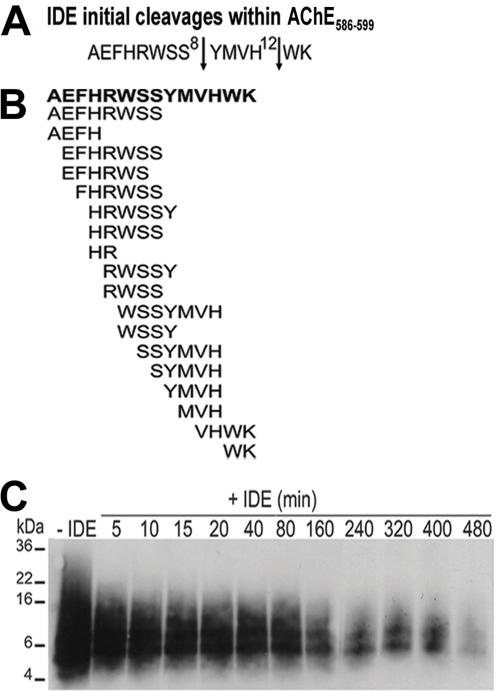
IDE degrades both monomeric and oligomeric forms of AChE_586-599_. (A) Positions of IDE initial cleavages (*arrows*) within AChE_586-599_. (B) Cleavage map after complete IDE digestion of AChE_586-599_. 63 µM AChE_586-599_ was incubated with or without 45 nM IDE (A) or 273 nM IDE (B)(37°C, 30 min) and RP-HPLC peaks analyzed by MS. (C) IDE degrades AChE_586-599_ oligomers. AChE_586-599_ oligomers (16 µM) cross-linked by photo-induced cross-linking were incubated with or without 16.3 nM IDE (37°C). Digestion products were resolved (16.5% Tris-Tricine SDS-PAGE), electro-blotted onto nitrocellulose and probed with Mab 105A (specific for AChE_586-599_ in β-sheet conformation). Marker proteins are indicated.

IDE has been proposed to be an amyloid scavenger recognizing structural β-rich folds found in amyloid forming peptides and has been shown to degrade soluble monomeric Aβ [4]. Since AChE_586-599_ is the only hAChE peptide reported to form amyloid fibrils, we investigated the capability of IDE to digest this peptide. IDE degraded AChE_586-599_ and the initial cleavage sites of IDE on this peptide were between Ser^8^-Tyr ^9^, and His^12^-Trp^13^ (Figure 3A). The identity of cleavage products is reported based upon AChE_586-599_ synthetic peptide numbering (Ala^1^ to Lys^14^)(Figure 3B). An initial IDE cleavage site was identical on both AChE_586-599_ (Ser^8^-Tyr^9^) and T40 (Ser^19^-Tyr^20^), suggesting that one of the dominant binding motifs for IDE can be found within the AChE_586-599_ portion of T40. Also, a significant proportion of generated products contained the YMVH motif of high propensity for β-sheet conversion.

Since IDE digests only monomeric forms of Aβ [35], [36], we examined the ability of IDE to degrade oligomeric AChE_586-599_. AChE_586-599_ oligomers were recognized by monoclonal antibody (Mab) 105A, demonstrating β-sheet conformation, and migrated mainly as a smear of SDS-stable oligomers (4-36 kDa) (Figure 3C, lane ‘-IDE’). In contrast to the lack of effect on Aβ oligomers, IDE degraded AChE_586-599_ oligomers (Figure 3C), in an insulin-sensitive manner (data not shown). Initially (5–80 min), IDE preferentially digested small and large oligomeric species (bottom and top of the smear). From 160 to 480 min, the 5 and 8 kDa species were partially degraded although persisted after 480 min incubation, suggesting resistance to IDE. These two oligomeric forms may correspond to trimers and pentamers according to their observed molecular weights (AChE_586-599_ being 1.86 kDa).

### NEP digests preferentially monomeric and oligomeric AChE_586-599_

**Figure 4:**
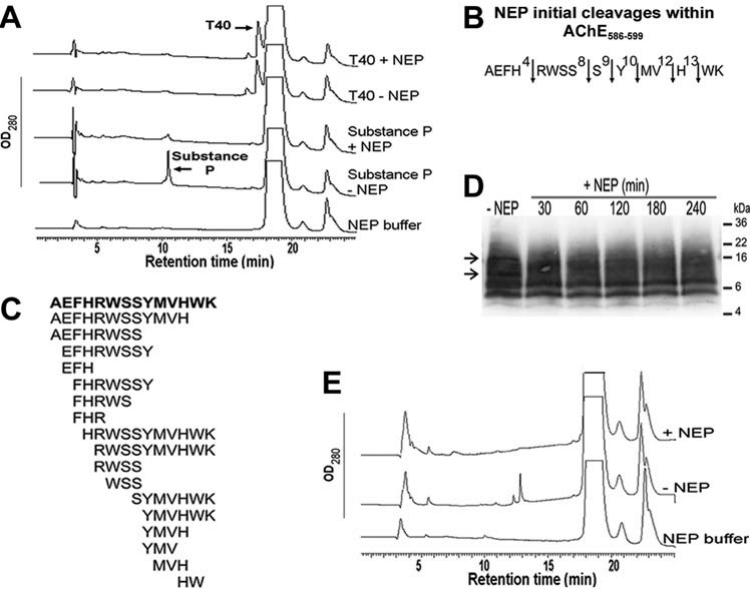
NEP preferentially degrades monomeric forms of AChE_586-599_. (A) 60 µM of either substance P or T40 were incubated with or without 1.2 µM NEP (37°C, 4 hours) and the digestion mixture subjected to RP-HPLC. (B) Positions of NEP initial cleavages (*arrows*) within AChE_586-599_. (C) Cleavage map after complete NEP digestion of AChE_586-599_. 40 µM AChE_586-599_ was incubated with 772 nM NEP (30 min (B) or 4 hours (C), 37°C) and RP-HPLC peaks analyzed by MS. (D) NEP degrades AChE_586-599_ oligomers. AChE_586-599_ oligomers (14.8 µM) cross-linked by photo-induced cross-linking were incubated with or without 285 nM NEP (37°C). Digestion products were analyzed as described in Figure 3C. A*rrows* indicate two digested AChE_586-599_ oligomers. (E) T40/IDE products are substrates for NEP. 60 µM T40 was incubated with 50 nM IDE (37°C, 30 min) and products separated by RP-HPLC. Peaks e–f (see Figure. 2C) were lyophilized, incubated with 772 nM NEP (37°C, 2 hours) and subjected to RP-HPLC.

Along with IDE, NEP is an important Aβ-degrading enzyme in the brain. The levels of non-amyloidogenic monomeric and dimeric forms of T40 remained unchanged after exposure to NEP (Figure 4A). The control degradation of substance P confirmed the presence of NEP activity under these assay conditions. NEP digests both monomeric and oligomeric forms of Aβ, hence it was appropriate to test its ability to degrade monomeric and oligomeric AChE_586-599_ [11]. NEP degraded AChE_586-599_ with complete digestion by 4 hours (Figures 4B and 4C). Although NEP targeted AChE_586-599_ more broadly than IDE, some of the cleavages were conserved between the two enzymes (Ser^8^-Tyr^9^ and His^12^-Trp^13^, AChE_586-599_ numbering). Some peptides were generated by both NEP and IDE (e.g. AEFHRWSS), however NEP also allowed some larger peptide species to remain intact (e.g. AEFHRWSSYMVH)(see Figures 3B and 4C).

NEP degradation of AChE_586-599_ oligomers was different and less efficient than IDE with a greater range of untargeted oligomers. Indeed, NEP only degraded the oligomer species at 10 and 16 kDa (Figure 4D, arrows), which remained intact during 240 min incubation in the absence of NEP (Figure 4D, lane ‘-NEP’). NEP-resistant 14–15 kDa species became apparent and might correspond to octamers according to their observed molecular weights. As previously observed for IDE, the 5 and 8 kDa species were resistant to digestion.

To assess the potential for interrelationships between IDE and NEP degradation, peaks e–f (see Figure 2C) from a 30 min IDE/T40 digest were subjected to NEP digestion for 2 hours. Peaks e–f were selected for the largest variety of CSR species among non-CSR related peptides. NEP was capable of degrading to completion the T40 products generated by IDE (Figure 4E).

### T40/IDE digestion triggers conformational changes

**Figure 5:**
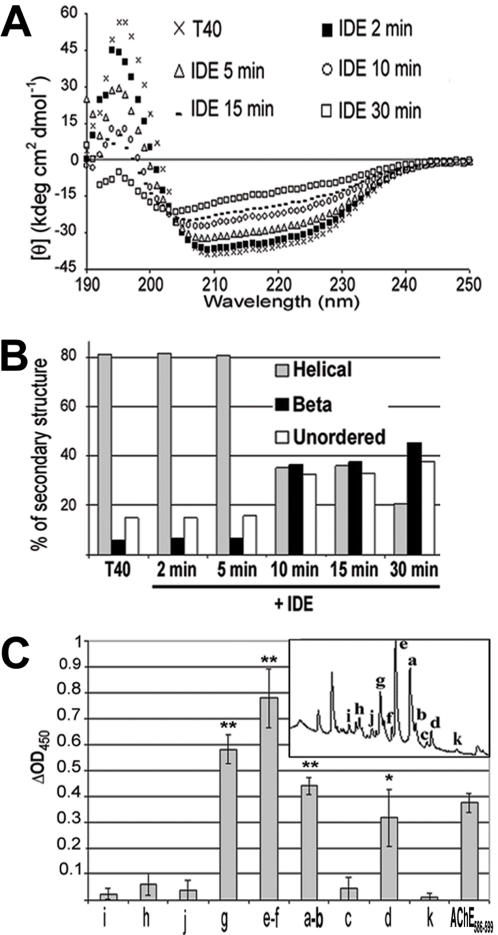
Conformation and surfactant properties of CSR species generated from T40 by IDE. (A) and (B) T40/IDE digestion triggers a switch to β-structure. T40 CD spectra (250 to 190 nm) before and after addition of IDE (A) and percentage of secondary structures (B). (C) CSR species are surface-active. T40/IDE digest (30 min) was subjected to RP-HPLC. Peaks (a–k, as annotated in the *inset*) were lyophilized, re-suspended in 200 mM sodium acetate pH 3 and surface tension measured before and after neutralization (1M NaH_2_PO_4_, pH7.2). Surface tension calculations were as described in Methods. ‘*’ p<0.05, and ‘**’ p<0.007.

Non-amyloidogenic T40 is α-helical either as a synthetic peptide or within hAChE [27], [28], [29], whereas AChE_586-599_ adopts a β-sheet conformation and self-assembles into amyloid fibrils [31], [30]. We have shown that some of the cleavage products generated from T40 by IDE contained CSR species encompassing motifs predicted to have a propensity for conversion to β-sheet (e.g. AEFHR and YMVHW). Therefore, we investigated the conformation of the T40/IDE digestion products. Circular dichroism (CD) spectra were determined during digestion of T40 by IDE at various times (Figure 5A). Prior to digestion, T40 displayed an α-helical spectrum with double minima at 209 and 222 nm. During IDE digestion, the ellipticity at 222 nm progressively decreased, suggesting a reduction in α-helices, accompanied by a shift of the minimum at 209 nm toward lower wavelengths that can be accounted by an increase of unordered structures (characterized by a single minimum below 200 nm). The negative ellipticity in the 210–220 nm indicated the presence of β-structures. After digestion, the spectrum intensity was reduced in the entire far-UV region, probably as the result of the formation of aggregates (see below). The total digest of T40 by IDE, as shown in Figures 2C and 2E, resulted in a mixture of peptides, not all of which necessarily adopted a β-sheet conformation. This could explain the complex nature of the CD spectrum observed during digestion, which did not show a fully β−sheet conformation. Therefore, to extract secondary structure components from the complex spectrum, we used spectral deconvolution utilizing an algorithm validated against the largest reported reference protein dataset [37]. Quantification of the secondary structures revealed that up to 10 min digestion, β-structure content remained negligible (5.9%), increasing substantially to 45.5% at 30 min, concomitant with a decrease in α-structure (from 81% to 20.5%) and an increase in unordered structure (from 14.9% to 37.4%)(Figure 5B). These temporal secondary structure changes correlated with the progressive accumulation of CSR species or their precursors peaking at 30 min (Figure 2E). When applied to three independent T40/IDE digests of 30 min, analysis of the CD spectra by deconvolution showed large and unambiguous changes with a decrease in α-helical content from 80.83±0.38% to 14.56±10.45% (p<0.008), an increase in β-structure from 6.90±0.89% to 53.70±15.34% (p<0.03), and an increase in unordered structure from 15.37±0.42% to 32.93±7.22%.

### T40/IDE generated CSR species are surface-active

At acidic pH, AChE_586-599_ remains monomeric and is not surface-active. In contrast, at neutral pH this peptide self-assembles into amyloid fibrils and reduces the surface tension of an air-water interface (measured by differential absorbance) in a similar manner to Aβ [38], [39]. The T40/IDE products a–k (Figure 5C *inset*) were analyzed for surface activity. Only peaks a, b, d, e, f and g showed an increase of ΔOD at neutral pH (p<0.05), which demonstrated their pH dependant surfactant properties (Figure 5C). This is comparable to a reduction of surface tension from ∼72 N.m^−2^ at low pH to ∼50 N.m^−2^ at neutral pH. All of these peaks contained CSR species. Products in peaks e–f, that contained the largest variety of CSR species including YMVHW (with high β-propensity) as a major species, had the biggest effect on surface tension upon neutralization. Peaks c and h were not surface-active at neutral pH, which may be explained by the very low levels of CSR species represented. For most of the surface-active products (peaks a, b, e, f and g), the effect was greater than seen with 50 µM AChE_586-599_. Peaks i, j and k did not contain CSR species and did not exhibit surfactant properties upon neutralization. Thus, some of the peptides generated by IDE digestion of T40 share with synthetic AChE_586-599_ the unusual characteristic that their surface tension effects are strongly pH dependent, which appears to be linked with the presence of CSR species. Moreover, these effects upon neutralization were not solely due to the presence of hydrophobic or aromatic amino acids in their sequences since some non surface-active peaks (e.g. peak c) contained as many or more hydrophobic and aromatic residues as some surface-active peaks (e.g peak a).

### T40/IDE digestion promotes Aβ fibrilization and amyloid protofibril formation

**Figure 6:**
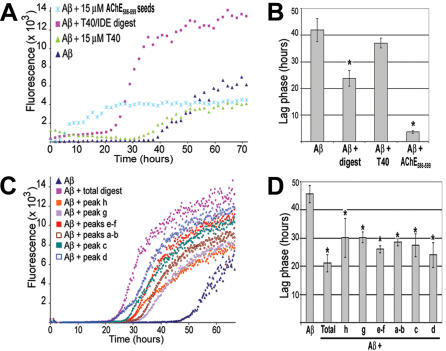
T40/IDE digestion products promote Aβ fibrilization. 15 µM Aβ was incubated with 165 µM ThT, with or without 15 µM T40, 15 µM AChE_586-599_ seeds, RP-HPLC isolated T40/IDE total digest or individual peaks (a–h, see Figure. 1C)(∼10 µM starting T40). Changes in ThT fluorescence were monitored (A and C) with the lag phase of Aβ fibrilization depicted (B and D). ‘*’ p<0.006 (B) and p<0.05 (D). Control experiments showed that there was no carry over of IDE activity in the T40/IDE digest under the sample preparation conditions (RP-HPLC and lyophilizations).

Having established that T40/IDE digestion produced CSR species, some of which possessed surfactant activity and changed conformation from α-helical to β-structures, we examined their ability as heterologous seeds to promote Aβ fibrilization. The quantity of Aβ fibrils was determined by changes in thioflavin T (ThT) fluorescence emission. For Aβ heterologous seeding, we used peptide seeds instead of monomers, experimental conditions that were identical to Diamant *et al* and were chosen for direct comparison with this previous study reporting the effect of hAChE on Aβ fibrilogenesis [40]. However, to preclude artifacts due to ThT binding to peptide oligomers/seeds themselves formed during IDE digests of T40, the values for peptide seed-ThT (no Aβ) were subtracted from all ThT assays (with Aβ). Thus, while it is possible that some experimental variation may be due to variable seed formation, these variations were accounted for in the statistical analysis of the experiment, which was based on three independent T40 digest experiments with replicates within each experiment. Aβ showed an increase in fluorescence after ∼42 hour nucleation process (lag phase)(Figures 6A and B). The addition of equimolar ratio of the non-amyloidogenic T40 (15 µM) to Aβ did not cause any changes in the lag phase or the apparent rate or the plateau height of Aβ fibril formation. This result confirmed that T40 did not affect fibrilogenesis even when it was present at a 1:1 ratio with the fibrilizing substrate and validated the use of T40 as a negative control. In contrast, equimolar ratio of AChE_586-599_ seeds (15 µM) to Aβ reproducibly reduced the Aβ lag phase by 11.6 fold (p<0.004), which confirmed the use of AChE_586-599_ as a positive control. Even at 1.5 µM (which represented 3.3% by mass of the total peptide), AChE_586-599_ peptide reduced the Aβ lag phase from 42 hours to 13±0.9 hours (p<0.006)(data not shown). The total T40/IDE digest (∼10 µM starting T40) also reproducibly reduced the Aβ lag phase by 1.8 fold (p<0.006). Moreover, the total T40/IDE digest also increased the apparent rate of fibril formation (from 48±15 for Aβ alone to 827±288 fluorescence units min^−1^)(p<0.043) and the plateau height (from 6493±930 for Aβ alone to 12333±1085 fluorescence units)(p<0.003). Each individual peak (a–h, see Figure 2C) from the T40/IDE digest also reduced the Aβ lag phase (p<0.005, p<0.05 for peak h) and some peaks increased the plateau height (p<0.012 for peaks e–f and p<0.0012 for peak d)(Figures 6C and D). Peaks g–h containing the fewest CSR species were the least efficient in lag phase reduction and plateau height increase. For the largest peptide fragments in any of the individual digest peaks, the maximum mass ratio of seed to soluble Aβ was 5.1% and for most peaks the mass ratio was lower. Thus, the measured kinetics show that reduced lag phase, increased rate and elevated plateau are all significant consequences of seeding with IDE digests of T40 (and that undigested T40 has no significant effect on any of these parameters). The mechanisms involved in the Aβ increase of both rate of fibrilization and plateau level after seeding with IDE digests of T40 are unknown. However, these results are entirely consistent with the reduction in lag phase and increase of both rate and plateau height observed when hAChE is present during Aβ fibrilogenesis [40].

**Figure 7:**
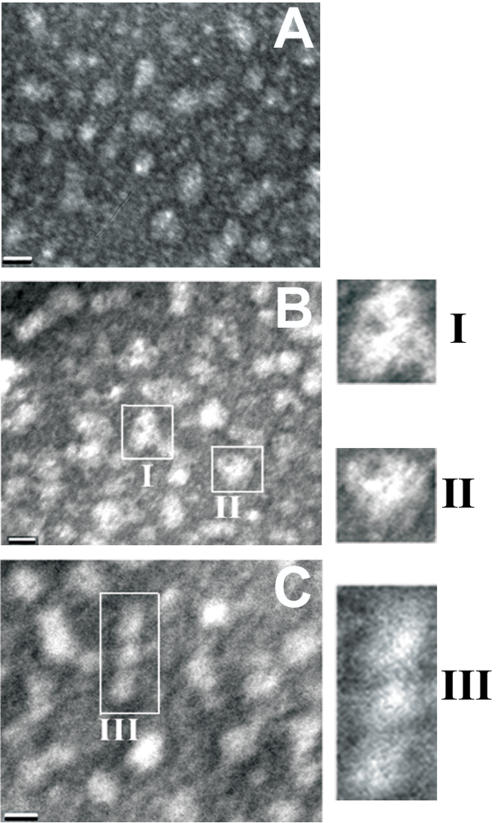
T40/IDE digestion products form amyloid protofibrils. Electron micrographs of negatively stained T40/IDE total digest showing spherical (A) annular (B, I–II) and beaded (C, III) protofibrils. The bar represents 10 nm.

The aggregation status of the total T40/IDE digest was examined by negative staining electron microscopy, which revealed predominantly spherical structures (diameter 4–14 nm)(Figure 7). Also observed were annular protofibrils (outer and inner diameters of 11 and 3 nm respectively), and “rods” (9 nm wide, 24–29 nm long) with some appearing as “beaded chains” composed of spherical sub-units. All these observations are consistent with the presence of amyloid precursors (oligomers)[41], [42], [43].

## Discussion

A number of proteases are involved in Aβ clearance in the brain, including two metalloproteases; IDE and NEP, with IDE digesting monomeric Aβ and NEP, oligomeric Aβ [10], [3], [4], [11]. hAChE promotes Aβ fibrilization and deposition in senile plaques but the hAChE domain involved remains uncertain [44], [22]. Therefore it is important to understand the mechanisms for the formation of hAChE amyloid species that could increase Aβ fibril formation during AD pathogenesis.

We have examined the ability of IDE and NEP to generate amyloid-forming species from the exposed and non-amyloidogenic hAChE T40 oligomerisation domain and the consequences for heterologous seeding of Aβ, a peptide thought to be the key player during AD. IDE and NEP may not be the only or major enzymes involved in the formation and/or clearance of hAChE species. Nonetheless, these two enzymes, which have already been implicated in turnover of amyloidogenic peptides, are present and active in the relevant compartment (namely the extracellular space of the brain) to attack an exposed part of a substrate (hAChE) that is also present and known to associate with plaques. hAChE T40 is exposed in the monomer and appears to remain vulnerable to proteolysis even in assembled tetramers [33], [32]. An outcome of such proteolytic attack may be hAChE fragments that are able to interact with Aβ, promoting fibril assembly. IDE degraded the non-amyloidogenic and α-helical T40, to generate CSR species, but also β-sheet forms of CSR. Upon binding to IDE, substrates undergo drastic conformational changes from α-helical to β-strands [45]. Thereafter, cleavage occurs at the β-strand sites [45]. Thus, the cleavage of the α-helical T40 by IDE implies that part of T40 is able to convert to β-conformation, which is supported by the identification within T40 of a unique predicted CSR (W_585_ to K_599_) with high propensity for conversion to non-native (hidden) β-strand [30]. Although IDE preference for cleavage is after aromatic and hydrophobic residues, multiple alignments for substrate binding can occur [46]. All IDE cleavage sites on T40 or AChE_586-599_ are consistent with the preferences previously described [46]. The T40/IDE cleavages indicate both distinct and secondary cleavages of an initial product. Indeed, CSR species terminating at Ser^19^ may have been generated by an initial cleavage at Ser^19^–Tyr^20^ of T40 followed by a second cleavage at the N-terminus. However, species encompassing the T40 N-terminus and terminating after Tyr^20^ may have been generated by distinct cleavage events. Both IDE and NEP appeared to digest primarily from the C-terminus of either T40 (IDE) or AChE_586-599_ peptide (IDE and NEP). Although NEP hydrolyses Aβ at specific sites, the enzyme digested AChE_586-599_ at almost every peptide bond in a non-specific manner. In contrast to Aβ, IDE acted on both monomeric and oligomeric AChE_586-599_ species (compared with only monomeric species for Aβ), whereas NEP acted mainly on monomeric species (compared with monomeric and oligomeric species for Aβ)[10], [11]. The fact that IDE was able to digest some oligomeric species of AChE_586-599_ is not inconceivable since the enzyme was shown to degrade substrate above 50 amino acids [47], [45]. However, such big substrates are less likely to be entrapped by IDE catalytic cleft and their degradation would be much slower. This could explain the ‘lack’ of efficiency of IDE towards some AChE_586-599_ oligomers and the relatively slow digestion process when compared to T40 or monomeric AChE_586-599._ Very few cleavage sites on Aβ and hAChE peptides are in common and are as follows; His^12^-Trp^13^ and Trp^13^-Lys^14^ for IDE, and Ser^8^-Tyr^9^ for NEP (AChE_586-599_ peptide numbering)(Figure S2 supporting information).

Significant differences were observed between the degradation capability of IDE and NEP. In contrast to NEP, IDE digested T40 and a bigger variety of AChE_586-599_ oligomers. However in conditions that allowed complete degradation of the monomeric AChE_586-599_ species, small AChE_586-599_ oligomeric species were slowly digested by IDE and untouched by NEP and some AChE_586-599_ oligomers were more resistant to degradation by both IDE and NEP. IDE was also more efficient (1 IDE:1200 peptides versus 1 NEP:52 peptides). One could postulate that IDE independently mediates the formation of CSR species and the clearance of soluble and some insoluble aggregates, whereas NEP could be involved in the clearance of the newly formed soluble CSR species. Indeed, we have demonstrated that CSR species generated from the T40 by IDE are a substrate for NEP. Therefore, if modification of hAChE by IDE occurs *in vivo*, both IDE and NEP deficiencies could alter the brain levels of CSR species and increase the risk of oligomerisation and fibril deposition. Indeed, reduced levels of these enzymes would still allow the formation of pathological species (albeit at a reduced rate) that could assemble into insoluble oligomers and fibrils, whereas clearance of oligomers (which is already inefficient when these enzymes are abundant) would be severely compromised. The small oligomer species resistant to both IDE and NEP could be initiating-agents in early pathological reactions. IDE activity was decreased in soluble fractions from the brain of AD patients compared to normal control brains [48], which suggests that a decrease in enzyme activity could be responsible for the increased accumulation of pathologic amyloid peptides during AD. It was also reported that in AD brain, IDE is less effective because it is oxidized [49]. NEP mRNA levels were reduced in amyloid affected areas of sporadic AD brain, which could be the cause of Aβ deposition [50].

IDE cleavage of the non-amyloidogenic T40 triggered a conformational change from α- to predominantly β-structure, a transition that was also observed for other amyloid proteins (e.g. insulin and prion protein)[51], [52]. Although native Aβ is unordered, α-helix formation is a key step for fibril assembly [53]. Several amyloid proteins, α-helical in the native state, contain stretches of α-helix in places that are predicted to form β-strand. These helices could form β-strands by unfolding into intermediates less likely to refold into a helical conformation [54]. Moreover, helical aggregates may convert short-range to long-range interactions triggering a β-transition [55]. Computational identification of non-native (hidden) β-strand propensity in protein sequences has predicted the minimal amyloidogenic fragments for Aβ and α-synuclein [30]. When applied to T40, two regions with a strong propensity for conversion to β-strand were recognized, YMVHWK the strongest and AEFHR more weakly (Figure 1), which is consistent with the fact that fragments (peaks e–f) containing these regions are the most surface-active. In the larger context of protein aggregation, our results support the importance of gatekeeper residues in preventing the conformational switch that leads to the formation of β-sheets and amyloid fibrils [56]. Evolutionary pressure may have sequestered CSR within T40 to maintain conformational integrity and to protect against deleterious misfolding.

Several intermediates of amyloid fibril formation have been identified with the first stage being spherical structural units that could associate to form beaded protofibrils from which fibrils nucleate and elongate [41], [43], [1]. Spherical oligomers from Aβ and α-synuclein specifically increase membrane conductivity [57]. Aβ (Arctic variant) and α-synuclein also form annular spheres resembling bacterial pore-forming toxins [42], [43]. The formation of pores in membranes may be one mechanism for the cytotoxicity seen in neurodegenerative diseases. In the case of Aβ, there is still controversy regarding the identity of the pathological species (monomers, small oligomers, large oligomers or fibrils) [58], [59]. However, several recent studies suggested that cognitive dysfunction correlates better with cortical levels of soluble oligomeric rather than insoluble (fibrilar) Aβ [60], [61], [62]. In our case, the surfactant and amyloidogenic CSR species generated by IDE might lead to an increased concentration of potentially toxic oligomeric forms, whether these are homo-oligomers of β-strand CSR species or hetero-oligomers also containing Aβ. The existence of such heterologous interactions is established in this study by the demonstration that the lag phase of Aβ assembly is reduced by the CSR species. However, the molecular details of the heterologous interaction remain to be characterized.

IDE catalysis generated CSR species that are highly dependent on pH for their surface-tension activity, which appeared to be solely the consequence of the presence of some CSR species and could not be explained by only the presence of hydrophobic or aromatic residues within the peptide sequences. In the case of Aβ, the surfactant properties were proposed to be detergent-like and linked to lysosomotropic activity resulting in accumulation of Aβ in lysosomes, release of lysosome contents and cell death [38]. AChE_586-599_ and CSR species from the T40/IDE digest were also surface-active. Thus, one could propose that both AChE_586-599_ and CSR species are detergent-like and could potentially permeabilize membranes. For AChE_586-599_, surfactant activity was directly linked to the threshold concentration for fibril formation [39], suggesting that surface-active T40 generated CSR species might also assemble into higher oligomeric species. Indeed, like other amyloid proteins, the T40/IDE digest formed amyloid protofibrils (spheres, annular spheres and “beaded rods”), which could contribute to neuronal toxicity in AD. Thus unlike the islet amyloid polypeptide for example, of which nested amyloidogenic peptides that formed fibrils were originated from an already amyloidogenic parent peptide [63], IDE digestion converted T40 (α-helical and non-amyloidogenic) into internal fragments that form β-sheet and are amyloidogenic.

hAChE was reported to increase Aβ fibrilization, an effect that was not mediated by isolated T40 [40]. Under the same experimental conditions, we confirmed that the non-amyloidogenic T40 does not promote Aβ fibril formation. In contrast, products generated from a T40/IDE digest and AChE_586-599_ seeded Aβ, an effect measured as a reduction in lag phase in a fibril formation assay (by 2 and 11 fold respectively) and the T40/IDE digest also increased the rate of Aβ fibril formation (by 17 fold). This result underlines the importance of the T40 CSR, which is included within the T40/IDE products and AChE_586-599_, and provides an insight into the identity of an AChE domain that may cooperate with Aβ during AD pathogenesis.

In conclusion, we have clearly demonstrated that IDE-dependent cleavage of the non-amyloidogenic hAChE oligomerisation domain leads to a conformational switch to β-structure and liberates surface-active peptides that assemble into amyloid protofibrils and seed the aggregation of hetero-oligomers comprised of Aβ and CSR species. Therefore, IDE-mediated formation of amyloidogenic hAChE fragments may provide useful targets for the identification of fibrilogenic hAChE-derived species in the brain, which has so far been impossible due to the lack of information about relevant hAChE species. While the CSR peptides themselves may offer a potential target in the struggle to prevent abnormal protein aggregation in the brain, our results suggest that simply increasing IDE and NEP activity may not be as beneficial as anticipated. Furthermore, the role of the non-amyloidogenic AChE T40 domain in heterologous seeding interactions may have to be re-appraised in light of the proteolytic events reported here. To our knowledge, this study represents the first evidence of heterologous amyloid seeding by a proteolytic fragment from another protein. Such seeding could represent a novel initial trigger for not only AD but also other neurodegenerative diseases sharing common characteristics, in which the abundance of the major amyloidogenic specie may not be the only important factor.

## Materials and Methods

### Synthetic peptides, inhibitors and antibodies

T40 and AChE_586-599_ were prepared as described [28]. Insulin, 1,10-phenanthroline and substance P were from Sigma-Aldrich (UK). Aβ_1-40_ (EZBiolab, USA) was dissolved in DMSO at 1.6 mM. Specific rabbit anti-T40 antiserum (KD69) was raised with T40 conjugated to keyhole limpet hemocyanin using standard procedures.

### Preparation of AChE_586-599_ oligomers

AChE_586-599_ oligomers were covalently cross-linked by photo-activation using the photo-induced cross-linking of unlabelled proteins (PICUP)[64]. In the dark, 0.3 nmoles of AChE_586-599_ and 94 µM tris-bipyridyl ruthenium salts in 500 mM NaH_2_PO_4_ buffer pH 7.2 were incubated with 1.9 mM ammonium persulfate (30 sec). The reaction mixture was exposed to light (50 W mercury arc lamp filtered via 5 cm H_2_O and a 400 nm UV blocking filter) for 1 sec, and quenched in the dark with 89 mM DTT before ultrafiltration (10 kDa filter) and dilution with H_2_O.

### IDE and NEP activity assays

IDE and NEP (Merck, UK) digestions were at 37°C in 100 mM KHPO_4_/KH_2_PO_4_ buffer pH 7.5 (buffer A) or 100 mM Tris-HCl pH 7.4, 1% Triton X-100 (buffer B) respectively. 16 µM T40 was incubated with 22 nM IDE or 309 nM NEP, and 16 or 14.8 µM AChE_586-599_ oligomers with 16.3 nM IDE or 285 nM NEP. Reactions were stopped by boiling in a Laemmli's dissociation buffer with 100 mM DTT and subjected to SDS-PAGE (10% Tris-Tricine for T40 and 16.5% Tris-Tricine for AChE_586-599_ oligomers).

To identify degradation products, 60 µM T40 was incubated with 50 nM IDE; 63 µM AChE_586-599_ with 45 nM or 273 nM IDE; and 40 µM AChE_586-599_ with 772 nM NEP and separated by reverse phase-HPLC (RP-HPLC). Peaks were collected and identities determined on a Q-TOF Micro mass spectrometer (Micromass, UK) with MassLynx 4.0 and MaxEnt 3 software. Lyophilized HPLC separated T40/IDE products were used for surface tension and seeding experiments.

60 µM substance P or T40 were incubated with or without 1.2 µM NEP (4 hours), stopped (0.5% trifluoroacetic acid) and subjected to RP-HPLC.

### RP-HPLC

Reaction products were resolved by RP-HPLC with a Sephasil C4 column (5 µm, 4.6×250 mm; Amersham Biosciences, UK) using a 5–95% linear gradient of acetonitrile in 0.1% trifluoroacetic acid over 25 min (flow rate 1 ml/min). The eluent was monitored by UV absorption at 280 nm.

### Western-blot

Nitrocellulose membranes were blocked with 5% (w/v) non-fat milk in PBS and incubated with KD69 anti-T40 antiserum or with the Mab 105A recognizing AChE_586-599_ in β-sheet conformation [28], followed by anti-rabbit or anti-mouse IgG conjugated to horseradish peroxidase (HRP). Products were visualized by enhanced chemiluminescence.

### Surface tension measurement

Analyses were performed in a 96-well plate format, as described [39]. Briefly, HPLC purified/lyophilized products were re-suspended in 80 µL 200 mM sodium acetate pH 3 and surface tension measured at 450 nm (BMG Polarstar plate reader) before and after neutralization (20 µL 1M NaH_2_PO_4_, pH7.2). ΔOD = (OD_offset position_ –OD_central position_)_neutral pH_–(OD_offset position_ –OD_central position_)_acidic pH_. At least three independent assays were performed and analyzed with the two-sample t-test.

### Circular dichroism

CD-spectra were recorded from 250 to 190 nm at 20°C in a quartz cuvette (1 mm path length) using a Jasco J-720 spectropolarimeter. The spectrum of 100 µM T40 in buffer A was recorded before addition of 83 nM IDE. The reaction mixture was incubated at 37°C for various times, cooled briefly on ice before spectrum recording. The mean spectra of multiple scans (scan speed of 50 nm min^−1^ and response time 4 sec) were deconvoluted with Selcon3 [37].

### Seeding experiments

HPLC separated/lyophilized T40/IDE products were treated as for surface tension measurement, and then re-lyophilized, re-suspended in buffer B and incubated for 2 hours at 37°C. Control experiments showed that there was no carry over of IDE activity under the sample preparation conditions (RP-HPLC and lyophilizations). The quantity of products was normalized to the height of the RP-HPLC peaks. AChE_586-599_ seeds were prepared by incubating 200 µM AChE_586-599_ in PBS for 3 hours under continuous agitation. Individual T40/IDE products or the total digest (∼10 µM starting T40), 15 µM AChE_586-599_ seeds and 15 µM T40 were dispensed in a 96-well plate (black wall, clear bottom; Greiner, UK) with 15 µM Aβ and 165 µM ThT in PBS. 15 µM Aβ in buffer B was used as a control for fibrilization. ThT fluorescence (excitation 450 nm, emission 480 nm) was measured at 37°C every 20 min, with 5 min shaking after every measurement, on a BMG Polarstar plate reader. The values of peptide-ThT were subtracted from the values of peptide-Aβ-ThT. At least three independent assays were performed and analyzed with the two-sample t-test.

### Electron microscopy

T40/IDE digest (30 min), as prepared for the seeding experiment, was adsorbed onto Formvar-coated 400 mesh copper grids, air dried, washed with distilled water, negatively stained with 2% aqueous uranyl acetate and viewed with a Zeiss Omega 912 microscope.

## Supporting Information

Figure S1 Cleavage map after complete digestion of T40 by IDE. 60 µM T40 was incubated with 50 nM IDE for 30 min at 37°C. The products were loaded onto a C4 reverse-phase HPLC column and separated using a 5–95% linear gradient of acetonitrile. HPLC product peaks were collected manually and their identities were determined by mass spectrometry. The full-length T40 sequence is shown at the top of the complete map. CSR species are shown in white letters on a black background.
(0.81 MB DOC)

Figure S2 Positions of IDE and NEP major cleavage sites within T40, AChE586-599 and Aβ^1-42^ sequences. (A) Positions of IDE major cleavage sites within T40, AChE586-599 and Aβ^1-42^ sequences. The cleavage sites of IDE within Aβ^1-42^ sequences were adapted from Mukherjee et al ((2000) J Neurosci 20: 8745-9). (B) Positions of NEP major cleavage sites within AChE586-599 and Aβ^1-42^ sequences. The cleavage sites of NEP within Aβ^1-42^ sequences were adapted from Carson et al ((2002) J Neurochem 81: 1-8). Gaps indicated by ‘-’ are introduced to maximise homology between T40 and Aβ^1-42^ sequences. Major cleavage sites are noted with arrows. Dashed arrows underneath the sequences represent cleavage sites occurring at a common peptide bond within hAChE peptides and Aβ^1-42^ sequences.
(0.11 MB DOC)
