# Supplementary material for: Heterologous Amyloid Seeding: Revisiting the Role of Acetylcholinesterase in Alzheimer's Disease
Source: PLoS One. 2007 Jul 25;2(7):e652. doi: 10.1371/journal.pone.0000652 (PMC1920558; doi:10.1371/journal.pone.0000652)

Supporting Information (available at [http://www.xxx](http://www.xxx/))

**Figure S1.** Cleavage map after complete digestion of T40 by IDE. 60 μM T40 was incubated with 50 nM IDE for 30 min at 37ºC. The products were loaded onto a C4 reverse-phase HPLC column and separated using a 5-95% linear gradient of acetonitrile. HPLC product peaks were collected manually and their identities were determined by mass spectrometry. The full-length T40 sequence is shown at the top of the complete map. CSR species are shown in white letters on a black background.


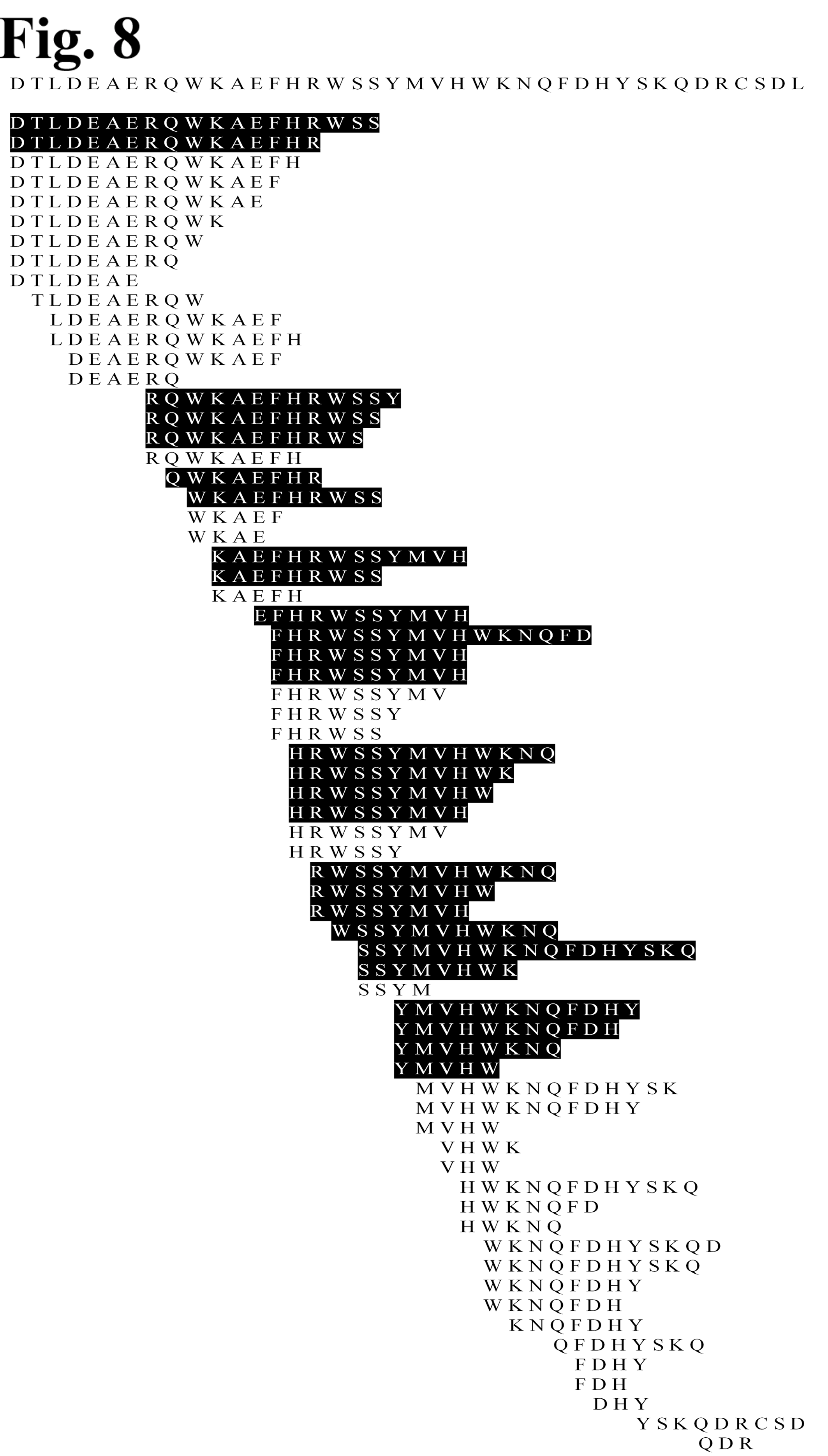

Supplement: Figure S1 — Cleavage map after complete digestion of T40 by IDE. 60 µM T40 was incubated with 50 nM IDE for 30 min at 37°C. The products were loaded onto a C4 reverse-phase HPLC column and separated using a 5–95% linear gradient of acetonitrile. HPLC product peaks were collected manually and their identities were determined by mass spectrometry. The full-length T40 sequence is shown at the top of the complete map. CSR species are shown in white letters on a black background. (0.81 MB DOC) [file pone.0000652.s001.doc]
