# Supplementary material for: Heterologous Amyloid Seeding: Revisiting the Role of Acetylcholinesterase in Alzheimer's Disease
Source: PLoS One. 2007 Jul 25;2(7):e652. doi: 10.1371/journal.pone.0000652 (PMC1920558; doi:10.1371/journal.pone.0000652)

Supporting Information (available at [http://www.xxx](http://www.xxx/))

**Figure S2.** Positions of IDE and NEP major cleavage sites within T40, AChE_586-599_ and Aβ_1-42_ sequences. (A) Positions of IDE major cleavage sites within T40, AChE_586-599_ and Aβ_1-42_ sequences. The cleavage sites of IDE within Aβ_1-42_ sequences were adapted from Mukherjee *et al* ((2000) J Neurosci 20**:** 8745-9). (B) Positions of NEP major cleavage sites within AChE_586-599_ and Aβ_1-42_ sequences. The cleavage sites of NEP within Aβ_1-42_ sequences were adapted from Carson *et al* ((2002) J Neurochem 81: 1-8). Gaps indicated by ‘-’ are introduced to maximise homology between T40 and Aβ_1-42_ sequences. Major cleavage sites are noted with *arrows*. *Dashed arrows* underneath the sequences represent cleavage sites occurring at a common peptide bond within hAChE peptides and Aβ_1-42_ sequences.


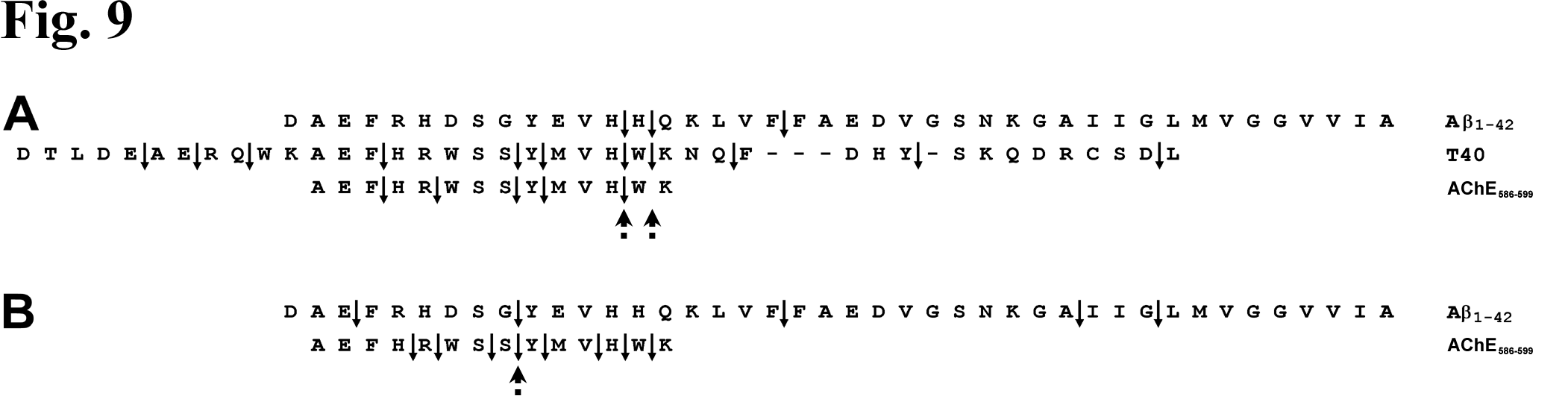

Supplement: Figure S2 — Positions of IDE and NEP major cleavage sites within T40, AChE586-599 and Aβ1-42 sequences. (A) Positions of IDE major cleavage sites within T40, AChE586-599 and Aβ1-42 sequences. The cleavage sites of IDE within Aβ1-42 sequences were adapted from Mukherjee et al ((2000) J Neurosci 20: 8745-9). (B) Positions of NEP major cleavage sites within AChE586-599 and Aβ1-42 sequences. The cleavage sites of NEP within Aβ1-42 sequences were adapted from Carson et al ((2002) J Neurochem 81: 1-8). Gaps indicated by ‘-’ are introduced to maximise homology between T40 and Aβ1-42 sequences. Major cleavage sites are noted with arrows. Dashed arrows underneath the sequences represent cleavage sites occurring at a common peptide bond within hAChE peptides and Aβ1-42 sequences. (0.11 MB DOC) [file pone.0000652.s002.doc]
